# Regarding the inference of causality in observational non-controlled studies. Comment on “Assessment of sexual function and its association with quality of life and disease severity in patients with atopic dermatitis” ‒ Reply^؆^

**DOI:** 10.1016/j.abd.2026.501451

**Published:** 2026-08-28

**Authors:** Aline Bressan, Carla Jorge Machado, Rita Fernanda Cortez de Almeida, Sueli Carneiro

**Affiliations:** a Department of Dermatology, Universidade do Estado do Rio de Janeiro, Rio de Janeiro, RJ, Brazil; b Department of Preventive and Social Medicine, Universidade Federal de Minas Gerais, Belo Horizonte, MG, Brazil

Dear Editor,

We appreciate the interest shown in our study and the pertinent observations presented by Marcato VR et al. Scientific debate is essential for methodological refinement and for the appropriate interpretation of findings.

Regarding the considerations about statistical power, we acknowledge that some analyses showed power below 20%, particularly in categorical comparisons stratified by sex. This aspect was explicitly described in the limitations section of the article in order to avoid overinterpretation of non-significant results. Nevertheless, we emphasize that the primary objective of the study was exploratory, aiming to identify patterns of association between sexual function, quality of life, and clinical severity of Atopic Dermatitis^1^ (AD) in a Brazilian population that remains underexplored from this perspective.

In this context, it is also important to clarify that the study design did not include a control group. This methodological decision was intentional, as the objective was not to compare patients with and without AD, but rather to understand the internal relationships between clinical severity, quality of life, and sexual function within the affected population itself. The inclusion of a control group would have addressed a different research question — namely, the absolute difference compared to the general population ‒ whereas our focus was to analyze intragroup association patterns. Furthermore, the use of instruments with previously established normative values, such as the EQ-5D-3L, allowed contextualization of the findings in relation to the Brazilian population, although without direct comparison within the same sampling design.

It is important to highlight that the differences observed between the mean sexual function scores (QS-F and QS-M)^2^, ^3^ demonstrated high statistical power (>90%), reinforcing the robustness of the finding of greater impairment in female sexual satisfaction compared with males. Likewise, the comparison between the sample’s EQ-5D-3L scores and Brazilian population norms showed a statistically significant difference with high power (0.99), supporting the conclusion that AD significantly impacts overall health perception.^4^

Regarding the correlations observed, particularly in males, we agree that the low statistical power limits the generalizability of these findings. However, we believe that reporting these results alongside explicit information on statistical power allows for a transparent and scientifically responsible interpretation. The inclusion of post-hoc power calculations for each analysis was a deliberate strategy to prevent unwarranted conclusions and to underscore the need for studies with larger sample sizes.

Additionally, we emphasize that the study provides relevant original contributions, including the concomitant use of validated sexual function instruments and the EQ-5D-3L, enabling estimation of health utility specific to Brazilian patients with AD. These data are particularly important in the context of cost-effectiveness analyses and public health decision-making.^5^

We fully agree that replication in larger, multicenter samples, and potentially with comparative groups, will help consolidate and expand the findings presented here. In this regard, we consider our study an initial step toward the systematic assessment of sexual health in patients with AD, encouraging future longitudinal investigations with greater statistical power.

## ORCID ID

Carla Jorge Machado: 0000-0002-6871-0709

Rita Fernanda Cortez de Almeida: 0000-0001-7904-998X

Sueli Carneiro: 0000-0001-7515-2365

## Research data availability

Does not apply.

## Financial support

None declared.

## Authors’ contributions

Aline Bressan: Conceptualized and administered the project; collected the data; developed the methodology; and wrote the original draft, as well as reviewed and edited the manuscript.

Carla Jorge Machado: Developed the methodology, analyzed and interpreted the data, and reviewed and edited the manuscript.

Rita Fernanda Cortez de Almeida: Reviewed and edited the manuscript.

Sueli Carneiro: Reviewed the manuscript and supervised the project.

All authors read and approved the final manuscript for publication.

## Conflicts of interest

None declared.
